# Investigation of mRNA expression changes associated with field exposure to DDTs in chickens from KwaZulu-Natal, South Africa

**DOI:** 10.1371/journal.pone.0204400

**Published:** 2018-10-11

**Authors:** Lesa A. Thompson, Yoshinori Ikenaka, Wageh S. Darwish, Yared B. Yohannes, Johan J. van Vuren, Victor Wepener, Nico J. Smit, Atnafu G. Assefa, Ahmed Tharwat, Walaa Fathy Saad Eldin, Shouta M. M. Nakayama, Hazuki Mizukawa, Mayumi Ishizuka

**Affiliations:** 1 Laboratory of Toxicology, Department of Environmental Veterinary Sciences, Graduate School of Veterinary Medicine, Hokkaido University, Sapporo, Hokkaido, Japan; 2 Water Research Group, Unit for Environmental Sciences and Management, North-West University, Potchefstroom, South Africa; 3 Food Control Department, Faculty of Veterinary Medicine, Zagazig University, Zagazig, Egypt; 4 Department of Chemistry, College of Natural and Computational Science, University of Gondar, Gondar, Ethiopia; 5 Educational Veterinary Hospital, Faculty of Veterinary Medicine, Zagazig University, Zagazig, Egypt; 6 Department of Environmental Veterinary Sciences, Graduate School of Veterinary Medicine, Hokkaido University, Sapporo, Hokkaido, Japan; Karolinska Institutet, SWEDEN

## Abstract

The objective of this study was to identify potential mRNA expression changes in chicken livers associated with environmental exposure to dichloro-diphenyl-trichloroethane (DDT) and its metabolites (DDTs). In particular, we focused on genes relating to the immune system and metabolism. We analyzed liver samples from free-ranging chickens in KwaZulu-Natal, South Africa, for contamination by DDTs. This area predominantly uses DDT in its malaria control program, and homes are sprayed annually with the pesticide. Genes relating to the immune system and metabolism were selected as potential genetic biomarkers that could be linked to higher contamination with DDTs. RT-qPCR analysis on 39 samples showed strong correlations between DDTs contamination and mRNA expression for the following genes: *AvBD1*, *AvBD2*, *AvBD6* and *AvBD7* (down-regulated), and *CYP17*, *ELOVL2* and *SQLE* (up-regulated). This study shows for the first time interesting and significant correlations between genetic material collected from environmentally-exposed chickens and mRNA expression of several genes involved in immunity and metabolism. These findings show the usefulness of analysis on field samples from a region with high levels of environmental contamination in detecting potential biomarkers of exposure. In particular, we observed clear effects from DDT contamination on mRNA expression of genes involved in immune suppression, endocrine-disrupting effects, and lipid dysregulation. These results are of interest in guiding future studies to further elucidate the pathways involved in and clinical importance of toxicity associated with DDT exposure from contaminated environments, to ascertain the health risk to livestock and any subsequent risks to food security for people.

## Introduction

The KwaZulu-Natal Province of South Africa is currently considered an endemic area for malaria and the mainstay of malaria control is the use of DDT in indoor residual spraying (IRS) programs [1,2]. Under guidance from the World Health Organization (WHO), local health centers annually spray the pesticide inside homes on walls and outside under roof eaves to reduce mosquito populations which transmit the disease. DDT and its breakdown metabolites (collectively known as DDTs) enter the environment as dust contamination and are a source of contamination via inhalation, contact and ingestion [3,4]. While exposure to DDTs is primarily via dermal contact and inhalation of aerosolized spray for workers administering DDT, ingestion is thought to be a more significant exposure route for other people and non-target species such as livestock [5–7].

When DDT was initially popular as an agricultural pesticide in the 1940s, it was thought to be toxic only to insects. However, by the 1960s it became apparent that non-target species were susceptible to toxic effects, notably DDT affecting reproduction in birds of prey populations, publicized widely in Rachel Carson’s book “Silent Spring” [8]. Eggshell thinning is the most well-known effect of DDT in birds, but other effects include reduced post-hatch survival, altered sexual behavior, neurotoxicity and smaller brain size [9–12]. Field sampling of avian species with lifelong environmental exposure has identified correlations between DDTs contamination and various hormonal and immune responses [13–16]. Chickens show signs of DDT toxicity, but higher levels compared to other avian species are required before clinical signs are seen [17–19]. Clinical signs reported in chickens after DDT exposure include tremors, hyperexcitability, death, and various reproductive effects (reduced egg production and hatchability, reduced perinatal survival, decreased eggshell thickness) [20–22]. The acute lethal toxicity dose in chickens is reported as >300 mg/kg [23]. Despite the growing list of toxic effects of DDTs, our understanding of the mechanisms of action is still lacking.

Xenobiotic contamination is not usually assessed in cases where an infectious agent is deemed the cause of death. Previous field studies have not investigated links between exposure with DDTs and immune function in chickens. Although reports have shown a link between DDT exposure and immunotoxicity in chickens experimentally [24–27], this study is the first to identify changes at the level of mRNA expression after environmental exposure. This evidence supports a need for further investigation of the role that DDTs may play in altering susceptibility to infectious agents. Plasma DDE levels have been linked to immune suppression in people [28]. Host defense peptides are conserved across a wide range of organisms, and play an important role in the innate immune system [29]. In birds, only beta-defensins (avian beta-defensins, AvBD, also known as gallinacins, GAL) have been described, with over 25 detected [30]. Although expressed in a wide range of tissues, expression of most *AvBD* genes is usually low in the liver [31]. Factors which affect expression include estrogen in the female reproductive tract, dietary Vitamin D_3_ concentration, inflammatory stimuli, and infections such as *Salmonella spp* and viruses [31–36]. Effects seen with infections depend on the organ affected, and also the chicken breed or age. No studies have previously linked these genes to DDTs exposure. However, any resulting alteration in immune function may increase a bird’s susceptibility to disease, leading to decreased productivity and also potentially an increased human health risk after consumption.

Similarly, metabolic changes caused by chemicals may also affect growth and productivity in chickens. Endocrine disrupting chemicals (EDCs) interfere with hormone systems, leading to disturbed glucose and fat metabolism [37]. Although risks have been documented in many species, understanding of the molecular mechanisms of action and involvement in metabolic disorders is still lacking. Organochlorine pesticides like DDT are known to be such EDCs, and interfere with hormone signalling and metabolic pathways [5,38,39]. Levels of DDTs have been linked to type 2 diabetes and metabolic syndromes in people [40,41]. Involvement of the insulin-like growth factor-binding protein 1 (IGFBP1) has been demonstrated in insulin signaling in chickens [42]. DDTs are lipophilic, with highest concentrations detected in high-lipid organs such as the liver. The liver is also a key location for whole body lipid metabolism, including fatty acid metabolism. Elongation of very long chain fatty acids elongase 2 (ELOVL2) is one of the two fatty acid elongase subtypes involved in polyunsaturated fatty acid (PUFA) biosynthesis in the chicken liver [43,44]. Squalene epoxidase (SQLE) is differentially expressed in fat tissues from fast-growing versus slow-growing chickens, and is involved in endogenous cellular cholesterol synthesis [45]. DDT exposure is associated with reproductive effects and gender alteration *in ovo*, and cytochrome P450 Family 17 Subfamily A Member 1 (CYP17A1) is involved in sex differentiation [46].

Chickens are an important food source for people, and thus any clinical or subclinical toxic effects could impact food security for local people where DDT is used to control vector-borne disease such as malaria. Chickens as livestock are relatively easy to sample and may be useful as a sentinel species for contamination by DDTs in wild birds in the region. The objective of this study was therefore to investigate mRNA expression changes associated with contamination by DDTs in free-ranging chickens from environmental exposure in an area where DDT is sprayed as part of a malaria control program. A number of genes were identified as biomarkers for DDTs exposure. RT-PCR was used to confirm statistical significance of dose-related changes in mRNA expression in a large number of samples across a broad range of contamination levels.

## Materials and methods

### Chemicals and reagents

Test reagents (oligo(dT) primers, reverse transcriptase (RT) buffer, and RT ACE) were purchased from Toyobo Co. (Osaka, Japan), TRI reagent from Sigma Chemical Co. (St. Louis, MO, USA), dNTP mix from Takara (Takara Bio Inc Japan), primer sets from Invitrogen (Carlsbad, CA, USA), and RNAlater from Sigma-Aldrich (St Louis, MO). A standard mixture of DDTs (Dr Ehrenstorfer GmbH, Germany) was purchased. Pesticide grade organic solvents and anhydrous sodium sulfate were purchased from Kanto Chemical Corp. (Tokyo, Japan). For microarray analysis, RNeasy Mini Kit from Qiagen (Hilden, Germany) was used, and Low Input Quick Amp Labeling Kit, Gene Expression Hybrization Kit, and Chicken (v2) Gene Expression Microarray (4X44K) were purchased from Agilent Technologies (Palo Alto, CA). All other reagents were purchased from Wako Pure Chemical Industries (Tokyo, Japan).

### Sampling sites and sample collection

**Fig 1:**
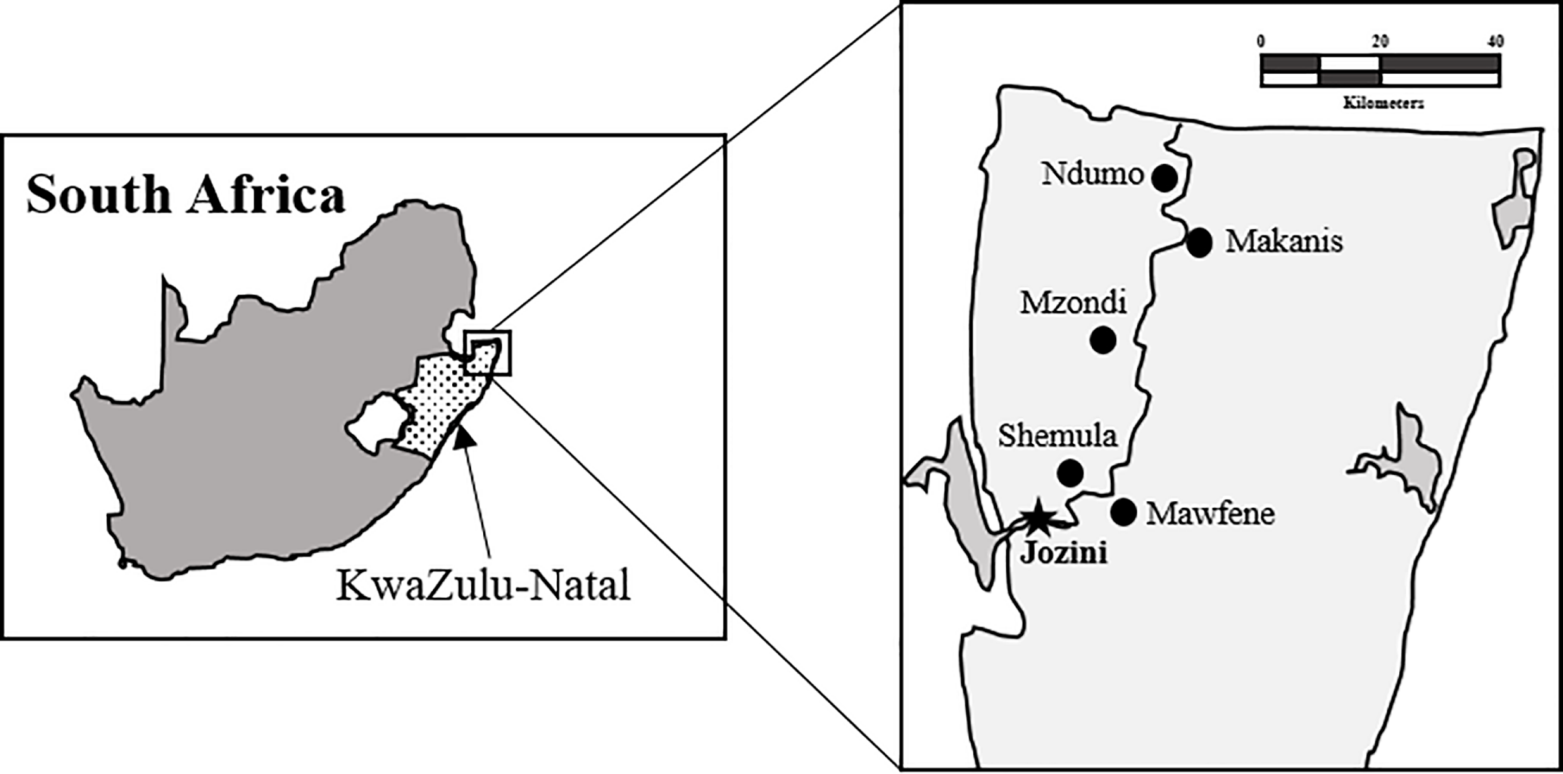
Map of region showing sampling sites in the northern part of KwaZulu-Natal Province, South Africa.

The sampling area was within the Jozini (town location 27° 25' 45.7" S 32° 3' 54.3" E) and Umhlabuyalingana (27° 12' 41.8" S 32° 26' 48.2" E) local municipalities, in Umkhanyakude District Municipality of KwaZulu-Natal Province of South Africa (Fig 1). Samples were collected from homesteads located in the following areas: Mamfene, Shemula, Mzondi, Makanis and Ndumo. At the time of sampling in October 2014, malaria was endemic in the region and DDT applied annually to homes. Chickens live free-range in homesteads where DDT is applied. Sampling was overseen by Jozini Health Center and the local veterinary office in Ndumo. Samples were obtained from domestic chickens with agreement from each homestead owner. Chickens were slaughtered by cervical dislocation followed immediately by decapitation and exsanguination. Liver samples were collected (n = 39) immediately after slaughter and aliquots stored in clean plastic vessels (for chemical analysis) and Eppendorf tubes containing RNAlater preservative (for mRNA expression analyses) (see Table 1 for details).

**Table 1:** Biometric data for chickens sampled in KwaZulu-Natal for this study.

	Mean	Range
Estimated age (months)^1^	13 ± 7	7–30
Weight (kg)^2^	1.5 ± 0.4	0.9–2.8
Body condition score^3^	2 ± 0.7	0–3
Lipid % in liver samples	3.8 ± 2.2	0.3–7.9
Supplied diet^4^	Maize (home-grown or shop-bought), leftovers, rice, bread, fresh vegetables	
Source	Shemula (11), Makanis (9), Ndumo (8), Mzondi (6), Mamfene (5)	

N/K = not known

1. Age estimation by owner at time of purchase.

2. Body weights for chickens are ante-mortem.

3. Based on Gregory and Robins 0–3 scale for layer hens [47].

4. Diet supplied by owner in addition to chickens foraging around the homestead.

This study was carried out in strict accordance with Hokkaido University guidelines, with veterinary certificates obtained from the agricultural office in Japan (Certificate number: 26 douken 523) and the veterinary office in Ndumo. Necessary approvals and international laws were adhered to regarding transfer of samples from South Africa to Japan.

### Sample preparation and storage

Liver was selected as the organ of interest for two main reasons. Firstly, DDT is lipophilic and is stored in body compartments with high lipid content, such as the liver. This organ is thus a good representation of contamination within the animal, and many studies analyze concentration of DDTs in the liver. Secondly, this organ is important in detoxification of chemicals and has many metabolic functions.

Liver samples were collected from freshly slaughtered chickens and stored via two methods. Samples for chemical analysis were frozen to -20°C shortly after collection. Samples for mRNA analysis were placed into RNAlater tissue storage reagent to stabilize and protect cellular RNA, and frozen. Samples were then transported to the Laboratory of Toxicology in Hokkaido University, and maintained at -80°C until analysis.

### Organochlorine extraction and analysis (DDTs)

Frozen samples were defrosted and analyzed to measure levels of DDT and its metabolites (*o*,*p’*-DDT, *p*,*p’*-DDT, *o*,*p’*-DDD, *p*,*p’*-DDD, *o*,*p’*-DDE and *p*,*p’*-DDE–collectively termed “DDTs”) using a slightly modified version of Yohannes et al.’s method [48]. Briefly, 1 g of liver was homogenized with anhydrous sodium sulfate before automatic extraction for 3.5 hours with a mixture of hexane:acetone (3:1 *v/v*) in a Soxhlet extractor (SOX416 macro SOXTHERM unit, Gerhardt, Germany). Each sample extract was spiked with 3,3’,4,4’-tetrachlorobiphenyl (PCB 77) surrogate standard, then concentrated prior to clean-up in a glass column packed with activated florisil and eluted with hexane : dichloromethane (7 : 3 *v/v*). After further concentration, 2,4,5,6-tetrachloro-*m*-xylene (TC*m*X) was added as a syringe spike, before analysis using a gas-chromatograph with ^63^Ni electron capture detector (GC-ECD: Shimadzu GC-2014, Kyoto, Japan). The machine condition parameters and QA/QC analysis were as in Thompson et al. [7].

### RNA extraction and cDNA synthesis

Total RNA was extracted using TRI reagent from the RNAlater-preserved samples, following the manufacturer’s protocol. Complementary DNA (cDNA) was synthesized according to Darwish et al.’s method [49].

### Selection of genes of interest

In our ongoing efforts to investigate the crosstalk between DDTs and mRNA expression relating to immunity and metabolism, we conducted a preliminary study to investigate gene expression changes using microarray. Total RNA samples were selected from two chickens matched for gender, body weight and body condition score. Neither chicken had any signs of ill health on ante-mortem or post-mortem examination; their liver total DDTs concentrations were 1,116.0 ng/g and 1,938.0 ng/g wet weight, respectively. A spectrophotometer (Nanodrop 1000, Thermo Fisher Scientific, Waltham, MA) and 2100 BioAnalyzer series II (Agilent Technologies, Palo Alto, CA) were used to determine nucleic acid quantity, quality and purity. Low Input Quick Amp Labeling Kit was used according to manufacturer directions, and cRNA purified using RNeasy Mini spin columns before quality inspection using the Agilent 2100 BioAnalyzer series II. Gene Expression Hybrization Kit was used to hybridize cyanine3-labeled cRNA. Samples were co-hybridized to Chicken (v2) Gene Expression Microarray (4X44K, Agilent Technologies), before incubation for 17 hours at 65°C. After washing, slides were scanned using an Agilent Technologies Microarray Scanner at 5 μm resolution. Raw data was digitized using Agilent Feature Extraction Software, version 10.7.3.1, and normalized to 75th percentile shift in GeneSpring (Agilent Technologies). The microarray dataset is accessible through ArrayExpress accession number E-MTAB-7130. This study assessed liver samples from chickens exposed environmentally to DDTs, and identified a number of functional areas and possible genes of interest. These genes fell into two categories: the innate immune system (*AvBD*s), and metabolism of steroid hormones and lipids (*IGFBP1*, *ELOVL2*, *SQLE*, and *CYP17A1*), with fold change expression differences between low and high DDTs-contaminated samples ranging from 8.3 to 21.5 for these genes.

### Quantitative real-time polymerase chain reaction

Chicken liver mRNA levels were determined by quantitative real-time RT-PCR using SYBR qPCR mix (Toyobo) and a StepOne real-time PCR system (Applied Biosystems). Primer sets for specific genes tested are described in Table 2. The method was performed according to Mureithi et al. [50]. In brief, PCRs were run with a final volume of 10 μl, containing SYBR qPCR Mix (Toyobo), 10 μM of each primer, 600 ng cDNA, 50X ROX reference dye and RNase-free water. Cycle conditions were as follows: 95°C for 20 s initial holding stage, 40 denaturation cycles of 95°C for 3 s and 62°C annealing for 30 s, and 95°C extension for 15 s. Amplification of a single amplicon of the expected size was confirmed using melting curve analysis and agarose gel electrophoresis. Experiments were repeated at least three times on different occasions. The sample containing the lowest contamination level of DDTs was assigned as a relative reference sample. In this study, two reference genes, namely *GAPDH* and *ACTB*, were used to normalize mRNA expression of target genes. Both reference gene mRNA expressions were not affected by exposure to DDT and were eventually expressed in all tested samples. Target gene expressions recorded in this study were normalized with respect to *GAPDH* mRNA expression and calculated relative to the nominal reference level using the comparative threshold cycle (Ct) method.

**Table 2:** qRT-PCR primer sequence information used in this study.

Gene		Sequence		Product size (bp)	Amplification efficiency (%)	Slope factor
	Accession number	Forward	Reverse			
*AvBD1 (GAL1)*	NM_204993.1	CCTGTGAAAACCCGGGACA	GCACAGAAGCCACTCTTTCG	145	92.24	-3.52
*AvBD2 (GAL2)*	NM_001201399.1	ACTGCCTGCCACATACATTTC	AGACAACCCTGGAGAAGCCT	127	98.76	-3.35
*AvBD6 (GAL6)*	NM_001001193.1	TTGCAGGTCAGCCCTACTTT	CCGGTAATATGGCCACCGAC	95	96.96	-3.40
*AvBD7 (GAL7)*	NM_001001194.1	ATTTCACATCCCAGCCGTGG	AGGCCTAGGAATGAAGGGCT	103	109.28	-3.12
*IGFBP1*	NM_001001294.1	TCACTGGATGGAGATTCCGC	AAGCTCCACAGAGAACCTGG	164	96.65	-3.41
*ELOVL2*	NM_001197308.1	CATGTGGGTTTCCCTTTGGC	GACTTCTGTTGTGACGGGGG	146	102.56	-3.26
*SQLE*	NM_001194927.1	CCATTTTTGGAGCGTCAGCC	GATGCCCAGGAAAGTCCACA	71	101.82	-3.28
*CYP17A1*	NM_001001901.2	CCCTACCTGGAGGCTACCAT	CGGACCAGAGGTTGATGACC	145	93.32	-3.49
*ACTB* (housekeeping)	L08165	CCCATCTATGAAGGCTACGC	TCCTTGATGTCACGCACAAT	152	100.59	-3.31
*GAPDH* (housekeeping)	NM_204305.1	ACACAGAAGACGGTGGATGG	GGCAGGTCAGGTCAACAACA	193	102.87	-3.26

### Statistical analysis

Amplification efficiencies for targets tested in this study ranged from 92.24% to 109.28%. These values were retrieved using slope factors from cDNA standard curves using the following formula: efficiency = -1+10(-1/slope), where efficiencies between 90 and 110% are typically acceptable [51]. Data analysis was conducted using Microsoft Excel 2014 and JMP Pro 12 (SAS Institute Inc., Cary, NC, USA). Contamination levels of DDTs are shown as median and range values in ng/g wet weight and ng/g lipid weight of tissue. Statistical significance was calculated using multivariate Spearman’s correlation analysis between mRNA expression and summed DDTs in chicken livers, by positive or negative correlation. A *p*-value of less than 0.05 was considered significant. Principle components analyses were used to investigate correlations of DDTs contamination, biometric data and mRNA expression. These analyses were made independently without data correction or adjustment of α value.

## Results and discussion

### DDTs concentrations

Contamination by DDT and its metabolites was detected in liver samples assessed (Table 3). The median of summed DDTs was 919 ng/g wet weight (ww), with a maximum of 14,398 ng/g ww. Concentrations of DDTs were comparable to those detected in chicken livers from Limpopo Province in another IRS-treated area of South Africa [52]. In the Limpopo study, the median sum of DDTs was 1,100 ng/g ww compared to 919 ng/g ww in this KwaZulu-Natal study. A study sampling chicken livers from an electrical and electronic waste (e-waste) site in China detected a much lower contamination level of 200 ng/g lw [53], compared to 29,235 ng/g lw in KwaZulu-Natal. DDT is not currently applied at the e-waste recycling area but legacy contamination is present.

**Table 3:** Levels of DDT and metabolites detected in liver from free-ranging chickens in KwaZulu-Natal.

	ng/g wet weight		ng/g lipid weight	
	Median	Range	Median	Range
*p*,*p’*-DDE	692	18–10,537	20,186	289–227,891
*o*,*p’*-DDD	10	<LOD—166	246	<LOD—5,919
*p*,*p’*-DDD	89	<LOD—1,840	2,929	<LOD—47,273
*o*,*p’*-DDT	11	<LOD—302	458	<LOD—8,280
*p*,*p’*-DDT	54	<LOD—1,923	1,333	<LOD—18,756
Sum of DDTs	919	36–14,398	29,235	555–288,928

<LOD = below limit of detection

The predominant congener was *p*,*p’*-DDE, and comprised approximately 75% of the DDTs. This congener has been linked to many toxic effects in birds, including eggshell thinning [54,55]. Estrogenic effects of DDTs are thought to affect avian embryos more than those of mammals, and phase I metabolites (DDE and DDD) may be more estrogenic than parent compounds [56–58].

Several factors may confound results from samples collected under field conditions–for example, difference in chicken breed, age, diet, body condition and health. As far as was possible, chickens selected from the study site were comparable. Husbandry methods resembled other households in the region. Adult birds of similar weight and body condition, without clinical signs of disease, were analyzed. No pathological conditions were detected on gross post-mortem examination of the birds. Chronicity of exposure may affect contamination levels, as bioaccumulation of DDTs occurs [59]. Comparison of DDTs concentration with biometric data (body condition score, body weight, estimated age and liver lipid percent) did not show any associations (data not shown). Statistical analysis did not show any difference in sampling location within the study area.

### qPCR mRNA expression results

Samples were all obtained from an area in KwaZulu-Natal Province where the Jozini Health Center administers DDT annually as part of their malaria control program. As this region is endemic for malaria, all homes are treated. In this scenario, it was not possible to obtain negative control samples from untreated homesteads and so statistical analyses were conducted by setting the reference sample for relative comparisons as that with the lowest concentration of summed DDTs.

**Fig 2:**
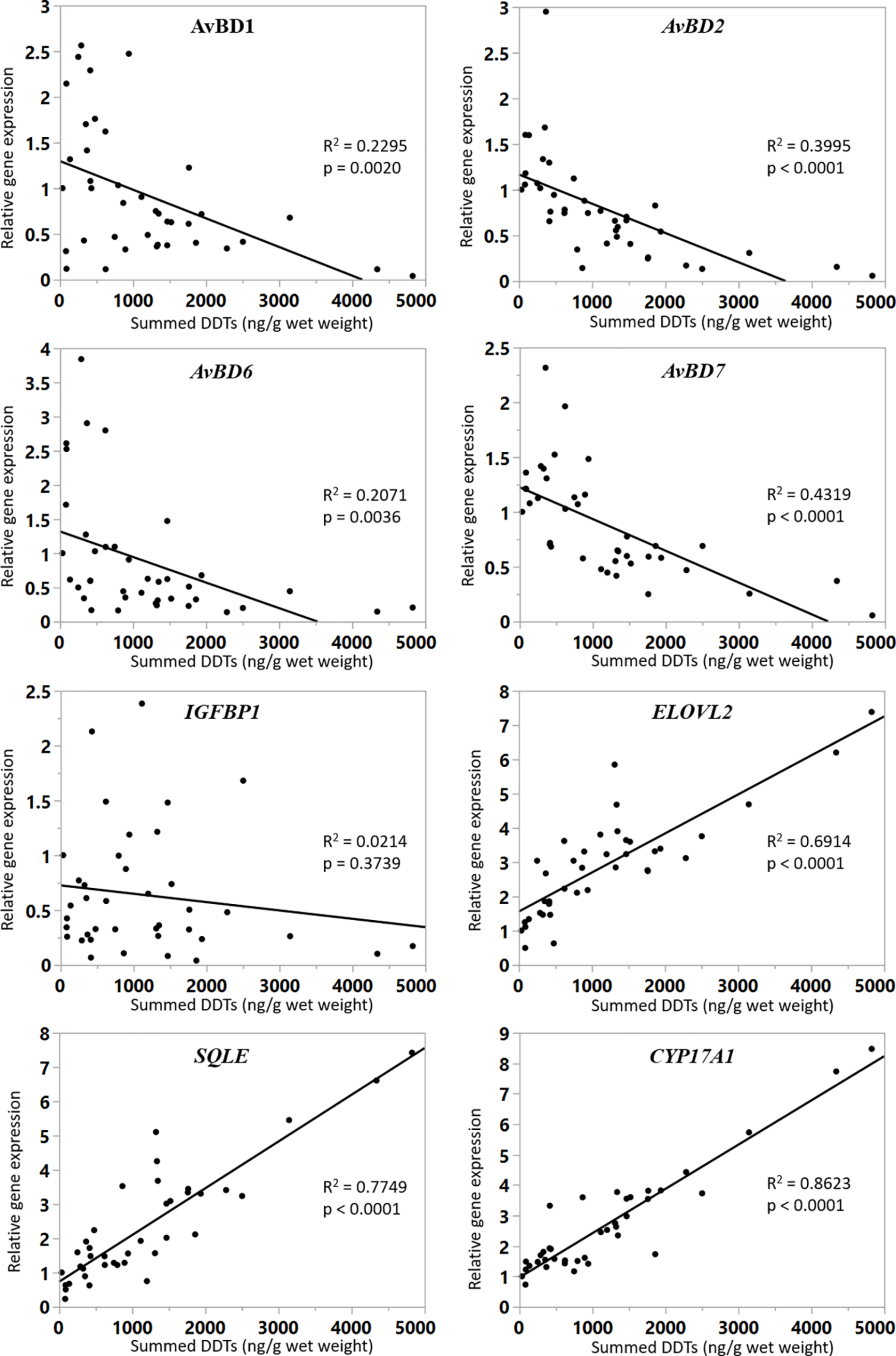
Correlation between mRNA expression and contamination levels (summed DDTs by wet weight). Summed DDTs (ng/g wet weight, x-axis) are plotted against relative mRNA expression (y-axis). R^2^ values and *p*-values are shown.

**Fig 3:**
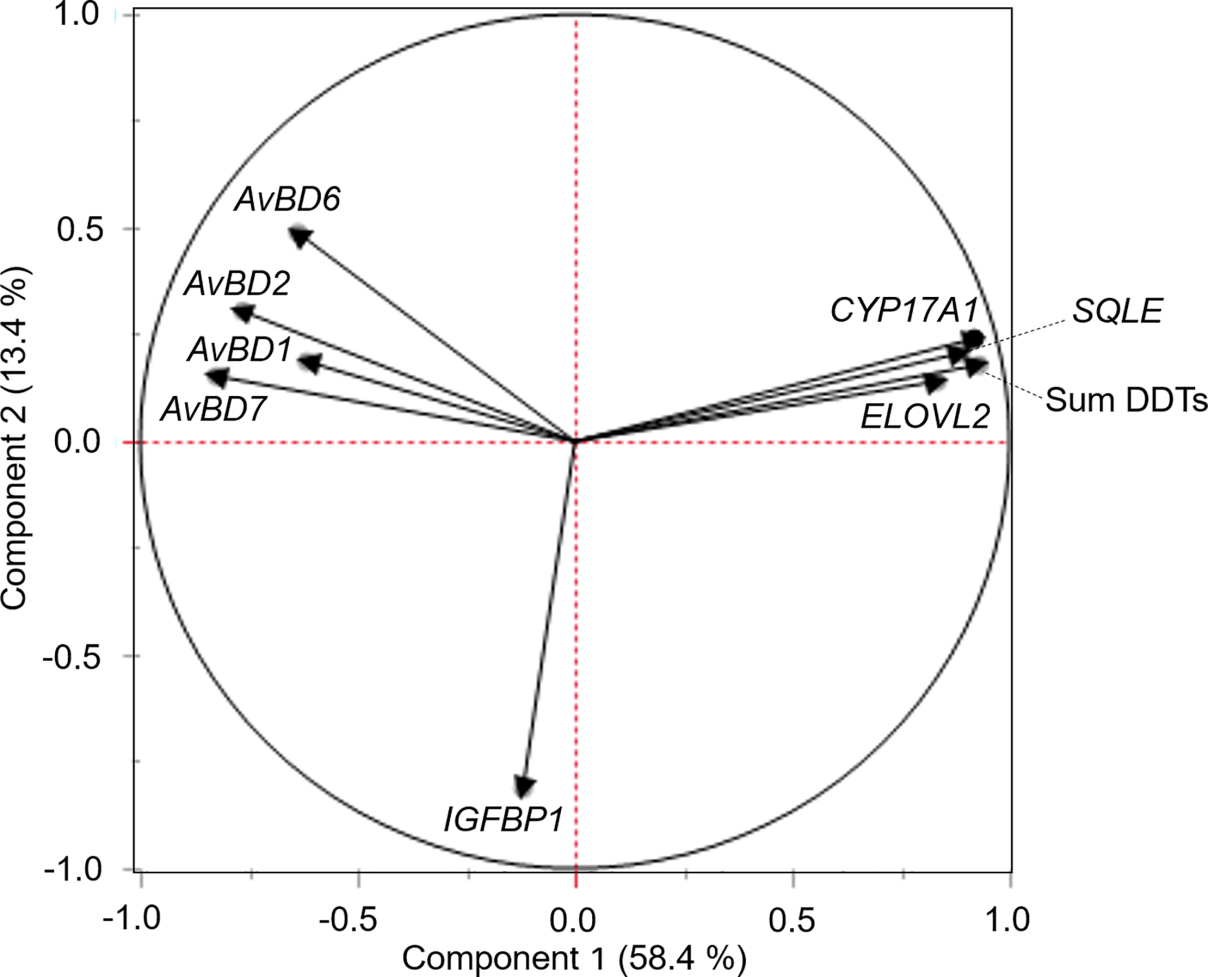
Principal components analysis of gene expression in chickens by summed DDTs concentrations. The first axis explains 58.4% of the variance in the data, showing strong negative correlation between DDTs contamination and beta-defensin mRNA expression, and strong positive correlation with mRNA expression relating to metabolism (*CYP17A1*, *ELOVL2* and *SQLE*). Key: *AvBD1* (avian beta-defensin 1), *AvBD2* (avian beta-defensin 2), *AvBD6* (avian beta-defensin 6), *AvBD7* (avian beta-defensin 7), *IGFBP1* (insulin-like growth factor-binding protein 1), *ELOVL2* (elongation of very long chain fatty acids elongase 2), *SQLE* (squalene epoxidase), *CYP17A1* (cytochrome P450 Family 17 Subfamily A Member 1).

A preliminary study using microarray analysis on samples from chickens exposed environmentally to DDTs identified a number of functional areas and possible genes of interest. Genes of interest were selected based on consideration of reported effects of DDTs and results from this microarray analysis. In particular, we focused on the innate immune system and on metabolism, particularly that of steroid hormones and lipids. A number of genes examined were significantly down-regulated (Fig 2): *AvBD1*, *AvBD2*, *AvBD6*, and *AvBD7*. Although there was a trend for down-regulation of *IGFBP1* with increasing DDTs, it was not a statistically significant association. The following genes were significantly up-regulated in samples with higher contamination levels of DDTs: *ELOVL2*, *SQLE* and *CYP17A1*. The principal components analysis plot (Fig 3) explains 71.8% of the variation between summed DDTs concentration and mRNA expression, with 58.4% on the first axis and 13.4% on the second. This plot suggests strong negative correlations between DDTs contamination and mRNA expression of avian beta-defensins assessed, and strong positive correlations with expression of *CYP17A1*, *ELOVL2* and *SQLE*.

Defensins have antimicrobial activity against many bacteria and fungi (reviewed in Cuperus et al [31]). They also play a role in immune regulation, binding to chemokine receptors, inducing pro-inflammatory cytokine expression, having anti-inflammatory properties and enhancing wound healing [60–62]. Expression of a range of *AvBD* genes showed a negative correlation with summed DDTs contamination: AvBD1 (*p* = 0.0020), *AvBD2* (*p* < 0.0001), *AvBD6* (*p* = 0.0036) and *AvBD7* (*p* < 0.0001). Reduction in expression levels of these important innate immunity genes may lead to increased susceptibility to disease with DDTs exposure. In this study, no signs of clinical disease were noted in the chickens ante-mortem or during post-mortem examination, but other investigations such as histopathology or bacterial culture were not performed.

IGFBP1 is a regulator of somatic growth and has been identified as a biomarker for visceral adiposity [63]. *IGFBP1* mRNA expression is involved in signaling between growth hormone (GH), thyroid hormone and body fat regulation in chickens but has not previously been linked to DDTs exposure [64]. Insulin signaling is affected by IGFBP1 in chickens [42]. There was a slight downward trend for *IGFBP1* expression with higher summed DDTs concentrations in the chicken livers but it was not statistically significant (*p* = 0.3739). Adipose tissue has been shown to be the primary tissue for storage of DDTs, and future work may elucidate a link between DDTs contamination and adipose production [65].

DDTs have been linked in mammalian species to obesity and metabolic syndromes [66]. Long chain PUFAs are necessary in vertebrates for normal growth and development, synthesized via either desaturation or elongation from dietary linoleic acid and a-linolenic acid. The *ELOVL2* gene plays a role in the fatty acid elongation pathway, and is essential for converting plant-derived α-linolenic acid (ALA) to eicosapentaenoic acid (EPA) and docosahexaenoic acid (DHA) [43,67]. *ELOVL2* expression was also significantly up-regulated with increasing DDTs contamination (*p* < 0.0001). There are no reports of metabolic syndromes such as diabetes occurring in birds due to DDT. However, this change in metabolism is important as poultry are an important source of long-chain polyunsaturated fatty acids (PUFAs) for people, particularly in countries where fish consumption is low [67].

Little is yet known about the function of SQLE. Administration of GH in rapidly-growing chickens down regulated expression of hepatic *SQLE*, which is also involved in lipid metabolism [64]. SQLE is involved in cholesterol synthesis in cells and also peripheral clock genes (delta 2 crystallin, Cry, and aryl hydrocarbon receptor nuclear translocator-like protein, Bmal) [68]. Cry and Bmal are involved in regulation of corticosteroid synthesis pathways, and their expression in broiler chicken adrenal glands is affected by ACTH treatment [69]. Again, expression of this gene, *SQLE*, was significantly up-regulated with increasing concentration of DDTs (*p* < 0.0001). Many xenobiotics are known to affect hormones, and this potential effect from DDTs on corticosteroid synthesis and cholesterol synthesis could affect many areas of metabolism. In livestock species, successful and rapid growth is particularly important, and any imbalances caused by xenobiotics are likely to affect food production. This could be significant in areas like KwaZulu-Natal where poverty and food security are problematic [70].

Studies on mammalian species have shown induction of cytochrome P450 (CYP) enzymes in rat liver microsomes after exposure with technical grade DDT [71]. CYP enzymes are important in phase I metabolism of xenobiotics [72]. Concentration responses to p,p’-DDT have been shown to vary between avian species [73]. CYP17A1 is also involved in androgen hormone synthesis and sex differentiation of birds [74–77]. The association between up-regulation of *CYP17A1* mRNA expression and DDTs concentration in sampled livers was highly significant (p < 0.0001). This strong correlation supports evidence linking DDTs exposure to biased sex ratios in gull embryos [46].

Regression analysis (Table 4) showed strong positive correlation between concentrations of *p*,*p’*-DDE, *p*,*p’*-DDD and *p*,*p’*-DDT with mRNA expression of *ELOVL2*, *SQLE* and *CYP17A1*. There was strong negative correlation between DDT congeners and expression of the *AvBD* genes assessed (*AvBD1*, *AvBD2*, *AvBD6* and *AvBD7*). For the *AvBD* genes, *p*,*p’*-DDE and *p*,*p’*-DDD were most significantly associated. *AvBD7* gene showed significant (*p*-value <0.0001–0.0391) negative correlation with all of the DDT congeners detected, and thus may be a sensitive biomarker for DDTs contamination. These data support the hypothesis that the *p*,*p’*-DDE congener, the most abundant contaminant and known endocrine disruptor, is the cause of many adverse effects associated with DDTs exposure. However, in light of the comparatively low concentrations of *p*,*p’*-DDD and *p*,*p’*-DDT in chickens sampled, it is interesting to note that these also significantly affect most of the genes analysed.

**Table 4:** Correlation between DDT congeners and mRNA expression in KwaZulu-Natal chicken livers. *A *p*-value of <0.05 was considered significant.

Gene	Regression statistics (R-squared (*p*-value))				
	*p*,*p’*-DDE	*o*,*p’*-DDD	*p*,*p’*-DDD	*o*,*p’*-DDT	*p*,*p’*-DDT
*AvBD1*	0.2473 (0.0013*)	0.0033 (0.7264)	0.0920 (0.0604)	0.0187 (0.4060)	0.0975 (0.0529)
*AvBD2*	0.3719 (<0.0001*)	0.1154 (0.0344)	0.3260 (0.0001*)	0.0550 (0.1507)	0.1478 (0.0157*)
*AvBD6*	0.1877 (0.0059*)	0.0522 (0.1617)	0.1711 (0.0089*)	0.0680 (0.1089)	0.0761 (0.0891)
*AvBD7*	0.3730 (<0.0001*)	0.1096 (0.0395*)	0.3866 (<0.0001*)	0.1101 (0.0391*)	0.2197 (0.0026*)
*IGFBP1*	0.0210 (0.3789)	0.0078 (0.5939)	0.0059 (0.6412)	0.0045 (0.6838)	0.0475 (0.1825)
*CYP17A1*	0.8298 (<0.0001*)	0.0589 (0.1367)	0.4179 (<0.0001*)	0.0420 (0.2105)	0.6816 (<0.0001*)
*ELOVL2*	0.6495 (<0.0001*)	0.0363 (0.2451)	0.2654 (0.0008*)	0.1928 (0.0052*)	0.6128 (<0.0001*)
*SQLE*	0.7318 (<0.0001*)	0.0570 (0.1432)	0.4516 (<0.0001*)	0.0240 (0.3464)	0.5894 (<0.0001*)

## Conclusions

This study shows for the first time interesting and significant correlations between genetic material collected from environmentally-exposed chickens and mRNA expression of several genes involved in immunity and metabolism. During malaria control programs, DDT has been applied to the study area in KwaZulu-Natal for more than a decade. This has led to a high level of environmental contamination and a source of DDTs for local livestock. The study clearly shows a link between this contamination of free-ranging chickens and mRNA expression changes that may have health impacts on both the chickens and the local human population.

Of particular interest are the genes involved in steroid synthesis, *CYP17A1* and *SQLE*. These are potential targets for the mechanism of estrogen-mimicry by DDTs. This endocrine disruption is well documented in several species. Up-regulation of the *ELOVL2* gene involved in fatty acid elongation is a strong link to lipid metabolism, and may help explain the connection between DDTs and metabolic syndromes reported in people. Down-regulation of several *AvBD* genes involved in the innate immune system are a potentially serious concern for the health of poultry livestock, where lowered immunity linked to increased infectious disease will impact not only bird health but also may be a problem for food security in people.

Ideally samples would be matched for confounding factors. It would also be useful to perform further chemical analyses to ascertain co-contamination with other xenobiotics in both the chickens and environment. An *in vivo* exposure study using environmental level concentrations of contaminants needs to be performed to remove potential confounding factors and bias such as age, breed, period of exposure, and concomitant exposure with other contaminants. It would be useful to consider DDTs across multiple generations to ascertain the full gamut of effects on the birds as embryos, developing young, and reproducing adults. Assessment of other closely related genes will also further elucidate the mechanisms involved and aid our understanding of the wide range of toxic effects and clinical importance of DDT and its metabolites.
